# Phosphorylation of the Transient Receptor Potential Ankyrin 1 by Cyclin-dependent Kinase 5 affects Chemo-nociception

**DOI:** 10.1038/s41598-018-19532-6

**Published:** 2018-01-19

**Authors:** Bradford E. Hall, Michaela Prochazkova, Matthew R. Sapio, Paul Minetos, Natalya Kurochkina, B. K. Binukumar, Niranjana D. Amin, Anita Terse, John Joseph, Stephen J. Raithel, Andrew J. Mannes, Harish C. Pant, Man-Kyo Chung, Michael J. Iadarola, Ashok B. Kulkarni

**Affiliations:** 1 Functional Genomics Section, National Institute of Dental and Craniofacial Research, National Institutes of Health, Bethesda, MD USA; 2 Department of Perioperative Medicine, Clinical Center, National Institutes of Health, Bethesda, MD USA; 3 The School of Theoretical Modeling, Washington, DC USA; 4 Institute of Genomics and Integrative Biology, New Delhi, India; 5 Neuronal Cytoskeletal Protein Regulation Section, National Institute of Neurological Disorders and Stroke, National Institutes of Health, Bethesda, MD USA; 6 University of Maryland, School of Dentistry, Baltimore, MD USA; 7 Present Address: Tulane University School of Medicine, New Orleans, LA USA

## Abstract

Cyclin-dependent kinase 5 (Cdk5) is a key neuronal kinase that is upregulated during inflammation, and can subsequently modulate sensitivity to nociceptive stimuli. We conducted an *in silico* screen for Cdk5 phosphorylation sites within proteins whose expression was enriched in nociceptors and identified the chemo-responsive ion channel Transient Receptor Potential Ankyrin 1 (TRPA1) as a possible Cdk5 substrate. Immunoprecipitated full length TRPA1 was shown to be phosphorylated by Cdk5 and this interaction was blocked by TFP5, an inhibitor that prevents activation of Cdk5. *In vitro* peptide-based kinase assay revealed that four of six TRPA1 Cdk5 consensus sites acted as substrates for Cdk5, and modeling of the ankyrin repeats disclosed that phosphorylation would occur at characteristic pockets within the (T/S)PLH motifs. Calcium imaging of trigeminal ganglion neurons from genetically engineered mice overexpressing or lacking the Cdk5 activator p35 displayed increased or decreased responsiveness, respectively, to stimulation with the TRPA1 agonist allylisothiocyanate (AITC). AITC-induced chemo-nociceptive behavior was also heightened *in vivo* in mice overexpressing p35 while being reduced in p35 knockout mice. Our findings demonstrate that TRPA1 is a substrate of Cdk5 and that Cdk5 activity is also able to modulate TRPA1 agonist-induced calcium influx and chemo-nociceptive behavioral responses.

## Introduction

Cyclin-dependent kinase 5 (Cdk5) is a proline-directed serine/threonine kinase that can modulate pain signaling^1,2^. Cdk5 is unlike other cyclin-dependent kinases because it is mostly active in post-mitotic neurons. Additionally, Cdk5 is not activated by a cyclin, but, instead, binds to two regulatory subunits, either p35 or p39, that are primarily restricted to neurons. Cdk5 activity is upregulated following peripheral administration of inflammatory agents such as carrageenan and Complete Freund’s Adjuvant (CFA)^1,3^. Increased Cdk5 activity can, in turn, promote both mechanical and heat hyperalgesia^1,2^. Cdk5 activity can also affect morphine tolerance, where reduced Cdk5 activity correlates with delayed tolerance^4^. Therapeutically, intrathecal injection of the Cdk5 inhibitor roscovitine appears to attenuate CFA-induced heat hyperalgesia but not mechanical allodynia^3^ while also reducing formalin-induced nociceptive behavior^5^. Cdk5 substrates include P/Q-type voltage-dependent calcium channels, which control calcium influx during nociception, and N-methyl-D-aspartate (NMDA) receptors, which relay pain signaling via the excitatory neurotransmitter glutamate^4^. Cdk5, however, is most directly linked to pain signaling by phosphorylating the pain transducing ion channel Transient Receptor Potential Vanilloid 1 (TRPV1). Cdk5 phosphorylates TRPV1 at T^407^, which, in turn, reduces channel desensitization^6,7^. Because of its role in modifying pain sensitivity, we wanted to screen for potential Cdk5 phosphorylation targets specifically enriched within pain-transmitting TRPV1^+^ nociceptors^8–11^. Our *in silico* search identified multiple potential macromolecular substrates, but the chemo-nociceptive channel Transient Receptor Potential Ankyrin 1 (TRPA1) was of particular interest for having 6 potential Cdk5 phosphorylation sites (T^101^, T^134^, S^242^, T^416^, S^449^, and T^485^) located within its intracellular N-terminal ankyrin repeat domain. TRPA1 is also of interest for being associated with inflammatory and neuropathic pain^12^ and for its genetic link to familial episodic pain syndrome^13^.

TRPA1 is expressed in peripheral C-fiber nociceptors^14^ where it is primarily regarded as a “chemo-sensor”^15^. TRPA1 is activated by not only natural pungent plant products (isothiocyanates in mustard oil, allicin in garlic, and cinnamaldehyde in cinnamon) but also by pollutants such as acrolein and formalin^15,16^. Endogenous activators of TRPA1 include the 4-hydroxynonenal aldehyde^17^ and the prostaglandin 15d-PGJ_2_ (15-deoxy-Δ-prostaglandin J_2_). These noxious chemicals are capable of binding to and activating TRPA1, which leads to acute pain. In addition, TRPA1 contributes to pathological pain and mediates mechanical and cold hyperalgesia^18–22^ following inflammation or nerve injury. Such pathological pain mediated by TRPA1 can be induced by multiple mechanisms. A variety of pro-inflammatory mediators^23–25^ or endogenous ligands^17,18^ can sensitize or activate TRPA1. Inflammation can cause upregulation of TRPA1 expression or sensitization of TRPA1 through phosphorylation via induction of downstream protein kinases^19,20,26^. Protein kinase A and Protein kinase C, for example, enhance activation of TRPA1 *in vitro*, which can then be attenuated by mutation of the prospective phosphorylated residues^27,28^. However, the mechanisms of TRPA1 sensitization through phosphorylation under pathophysiological conditions are largely unclear.

Within TRPA1, all six potential Cdk5 phosphorylation sites are situated in the N-terminal cytoplasmic domain. All of the typical consensus Cdk5 sites of (S/T)PX(K/H/R)^29^ in TRPA1 essentially overlap with a T/SxxH motif that is found in about 41% of ankyrin repeats^30^. Ankyrin repeats mainly contain a stereotypic motif composed of a pair of α-helices, a finger loop, and a β-hairpin. The T/SxxH tetrapeptide located in the ankyrin repeats aids in establishing the helix–turn–helix conformation in the ankyrin repeat while also promoting intra-molecular hydrogen bonds to confer conformational stability. Ankyrin repeats are thought to be involved in protein-protein interactions, but have also been suspected to display elastic qualities^31^. A unique feature that distinguishes TRPA1 from other TRP channels is the large number of cytoplasmic ankyrin repeats. The N-terminal domain of TRPA1 contains a total of 17 ankyrin repeats that are thought to confer sensitivity to chemical irritants^32^ and are believed to be essential for plasma membrane localization^33^. Another feature within the N-terminal cytoplasmic domain is a number of reactive cysteines (Cys^415^, Cys^422^, and Cys^622^ in the mouse TRPA1)^34^ that are thought to covalently bond to electrophilic compounds, thereby allowing TRPA1 to respond to multiple chemical irritants.

In order to determine if TRPA1 was a substrate of Cdk5, we immunoprecipitated a FLAG-tagged version TRPA1 for testing in an *in vitro* kinase assay. The immunopreciptiated TRPA1 was phosphorylated by Cdk5 and this modification could be blocked using an inhibitory peptide that disrupts Cdk5/p35 interactions^35^. To identify which of the 6 candidate sites were most likely phosphorylated by Cdk5, we screened 10 residue amino acid peptides containing the Cdk5 consensus sequence in an *in vitro* kinase assay and determined that 4 of the 6 potential Cdk5 phosphorylation motifs were highly phosphorylated. Calcium imaging studies in cultured trigeminal ganglion (TG) neurons from mice with genetically altered Cdk5 activity showed that Cdk5 enzymatic activity modulated the number of responsive neurons to the TRPA1 agonist allylisothiocyanate (AITC). Lastly, mice with increased Cdk5 activity had increased aversion to consumption of AITC, while the converse was true for mice with decreased Cdk5 activity. These data are consistent with the idea that Cdk5 can modulate noci-responsive ion channel activity of primary afferent neurons through post-translational phosphorylation with subsequent change in the sensitivity of the nociceptive neurons to multiple pain modalities.

## Results

### Bioinformatic Search for Potential Cdk5 Substrates in Nociceptive Neurons

Because of the key role of Cdk5 in modulating neuronal cytoskeletal dynamics, neurotransmitter release and pain signaling, we and others have searched for new Cdk5 targets either with mass spectroscopy or through a position scoring analysis^29,36,37^. While Cdk5 is highly expressed in all neuronal tissue, most identified candidate substrates have not been shown to be directly applicable to nociception, even though Cdk5 is a major regulator of pain signaling^1^. Within the dorsal root ganglia (DRG), Cdk5 is uniformly expressed throughout all types of sensory neurons^10^. The Cdk5 activator p35 is also expressed regularly throughout all DRG neurons, suggesting that further restriction of targets to the nociceptive subpopulation^9^ would be more informative. To specifically identify pain-related Cdk5 substrates, we narrowed our search of candidates to a nociceptive subset of neurons. This subset can be broadly identified by genetic labeling of the TRPV1-lineage since TRPV1 expression precedes full developmental differentiation of the various nociceptive subpopulations. We cross-referenced a RNA-Seq database of genes highly expressed in the TRPV1-lineage (TRPV1_L_) for proteins that contain the typical (S/T)PX(K/H/R) phosphorylation motif that is found in 49% of Cdk5 substrates^10,29^ (Supplementary Table 1). Proteins with Cdk5 sites located within extracellular or transmembrane domains were excluded as we focused on potential phosphorylation sites that are accessible to either membrane bound Cdk5/p35 or cytoplasmic Cdk5/p25. Examples of potential protein Cdk5 substrates with confirmed mass spectrometry or other published evidence of phosphorylation are presented in Supplementary Table 1. Our screen identified several voltage-gated channels (CACNA1I, KCNG2, KCNC2, KCNH6, SCN11A), particularly potassium channels, as potential Cdk5 substrates, suggesting the importance of this enzyme as a regulator of membrane potential and neuronal excitability. We also identified a conserved Cdk5 site in EGR1, EGR2 and EGR3 which suggests all three paralogs could be substrates of Cdk5, yet a Cdk5 motif is not conserved in EGR4. Three thermosensitive members of the transient receptor potential family were identified in our screen as well: TRPV1, TRPV3, and TRPA1 (Supplementary Table 1). TRPV1 is already a known Cdk5 substrate^6^. In this study, we focused on TRPA1, based on several reasons: First, we saw that TRPA1 has several putative Cdk5 sites located within ankyrin repeat domains at the N-terminal domain. Second, TRPA1 is a well-known transducer of noxious stimuli in primary afferent sensory neurons^15^. Third, the expression of TRPA1 is relatively high and enriched in TRPV1_L_ neurons, a population of neurons where Cdk5 is definitely known to be active and important for regulating TRPV1-mediated calcium influx^7,10^ (Supplementary Figure 1).

### Protein Modeling of the TRPA1 Ankyrin Repeats

**Figure 1:**
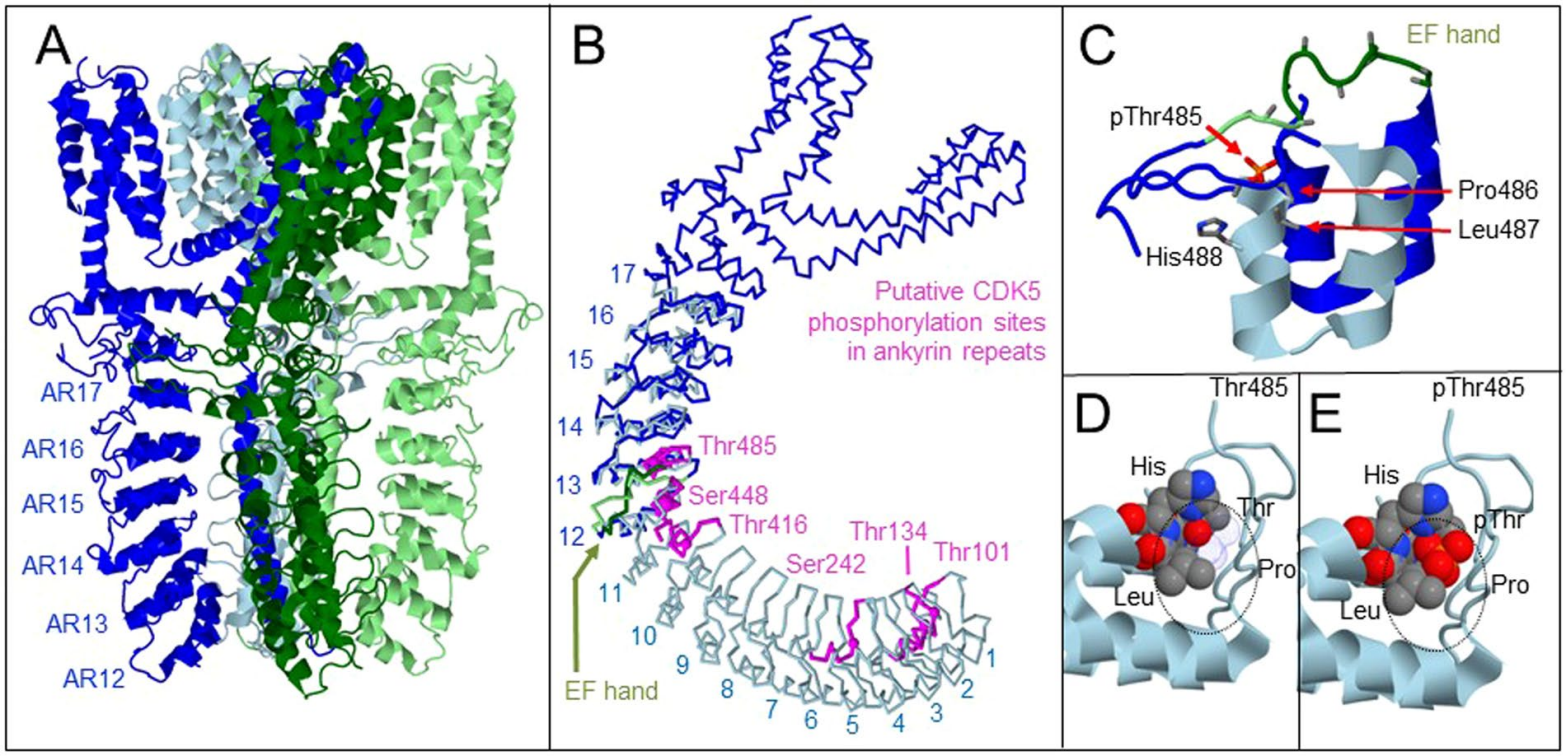
Protein model of TRPA1 ankyrin repeat domains with putative phosphorylation sites. TRPA1 is a large homotetrameric pore-forming channel. (**A**) cryo-EM was used to solve the structure of ankyrin repeat (AR) domains from AR12 all the way through the 6 transmembrane domains. (**B**) One member of the homotetrameric complex is shown including the locations of all 6 putative Cdk5 phosphorylation sites (pink) and the EF hand domain (green). ARs 1–11 (light blue) were modeled separately. (**C**) At Thr485, an empty pocket can be observed adjacent to the EF hand. Structural modeling (**D**,**E**) suggests this binding pocket is approximately the size of a phospho-threonine (pThr) residue, suggesting that the side chain of this Thr residue, once phosphorylated, would fill the pocket.

TRP channels have been implicated in sensory physiology to detect environmental cues such as temperature, light, and chemicals, and several members of the TRP family are known to have ankyrin repeats (AR) in their N-terminal domains^38,39^. TRPA1 contains 17 ankyrin repeats, whereas TRPV1, a known Cdk5 substrate, contains only 6. A total of 6 Cdk5 phosphorylation consensus sites were identified within the mouse TRPA1 ankyrin repeats (AR2 [T^101^], AR3 [T^134^], AR6 [S^242^], AR11 [T^416^], AR12 [S^449^], and AR13 [T^485^]). All of the potential Cdk5 sites in TRPA1 essentially consist of a T/SxxH tetrapeptide motif that is common to 41% of ankyrin repeats^30^. The structure of TRPA1 has recently been modeled using single-particle cryo-electron microscopy (CryoEM), and this three-dimensional structural data was used to determine if these T/SxxH containing ankyrin repeats within TRPA1 were structurally permissive as phosphorylation sites for Cdk5^40^. Because the TRPA1 CryoEM structure lacks information on ankyrin repeat domains 1 to 11, we constructed a model of these ankyrin repeats (Fig. 1A–C) linked to the resolved structure of the remaining N-terminal domain followed by the pore region and C-terminal. As revealed by the CryoEM structure^40^, the ankyrin repeats of TRPA1 exhibit compact and extended conformations. The noncanonical extended conformation is more consistent with the active site geometry of Cdk5 than a canonical helical conformation. With at least two distinct conformations to the ankyrin repeats of TRPA1, we wanted to model the full N-terminal domain to better assess exposure of the phosphorylation sites to solvents and provide evidence that a conformational transition is necessary to make the Thr/Ser sites accessible to the kinase. Since there are six potential phosphorylation sites, the model of the complete ankyrin repeats domain also shows the spatial arrangement and positioning of all potential Cdk5 phosphosites in relationship to other functionally important structural components including a reported EF hand-like domain (N^469^DISDTRLLNEGDL^481^ in the mouse TRPA1)^41^, noxious chemical accomodating sites, reactive cysteines, and the structurally conserved consensus ankyrin repeat sequence.

After developing the N-terminal of TRPA1 model, we decided to focus on the potential T^485^ phosphorylation site in further detail (Fig. 1C–E). In the CryoEM structure of TRPA1, the threonine hydroxyl group of T^485^ is 4 Å away from histidine whereas the most frequently observed threonine conformation at this site within the ankyrin repeats of TRPA1 is compact and hydrogen bonded to the histidine. The arrangement of T^485^ within the TxxH motif of AR13 suggests that this site is subject to conformational transitions that could contribute to the specific site recognition and multiprotein assembly^42,43^ necessary for phosphorylation by Cdk5. Modeling of AR13 region further revealed a cavity approximately the size of an additional phosphate group (Fig. 1D), which is where the phosphorylation event is modeled to occur (Fig. 1E). In the nonphosphorylated state, the Thr/Ser side chain makes a hydrogen bond with the histidine. Phosphate addition leads to rearrangement of this contact. When phosphorylated, the Thr/Ser phosphogroup is a potential binding site for partner proteins or peptides within these proteins. When compared to next homologous ankyrin repeats in AnkB, it is interesting to note that the potential Cdk5 sites of TRPA1 may be strategically situated in close proximity to important amino acid residues involved in protein binding (Supplementary Fig. 2). Therefore, one possible role of phosphorylation could be rearrangement of ankyrin-peptide interactions.

### Phosphorylation of Potential TRPA1 Sites by Cdk5

**Figure 2:**
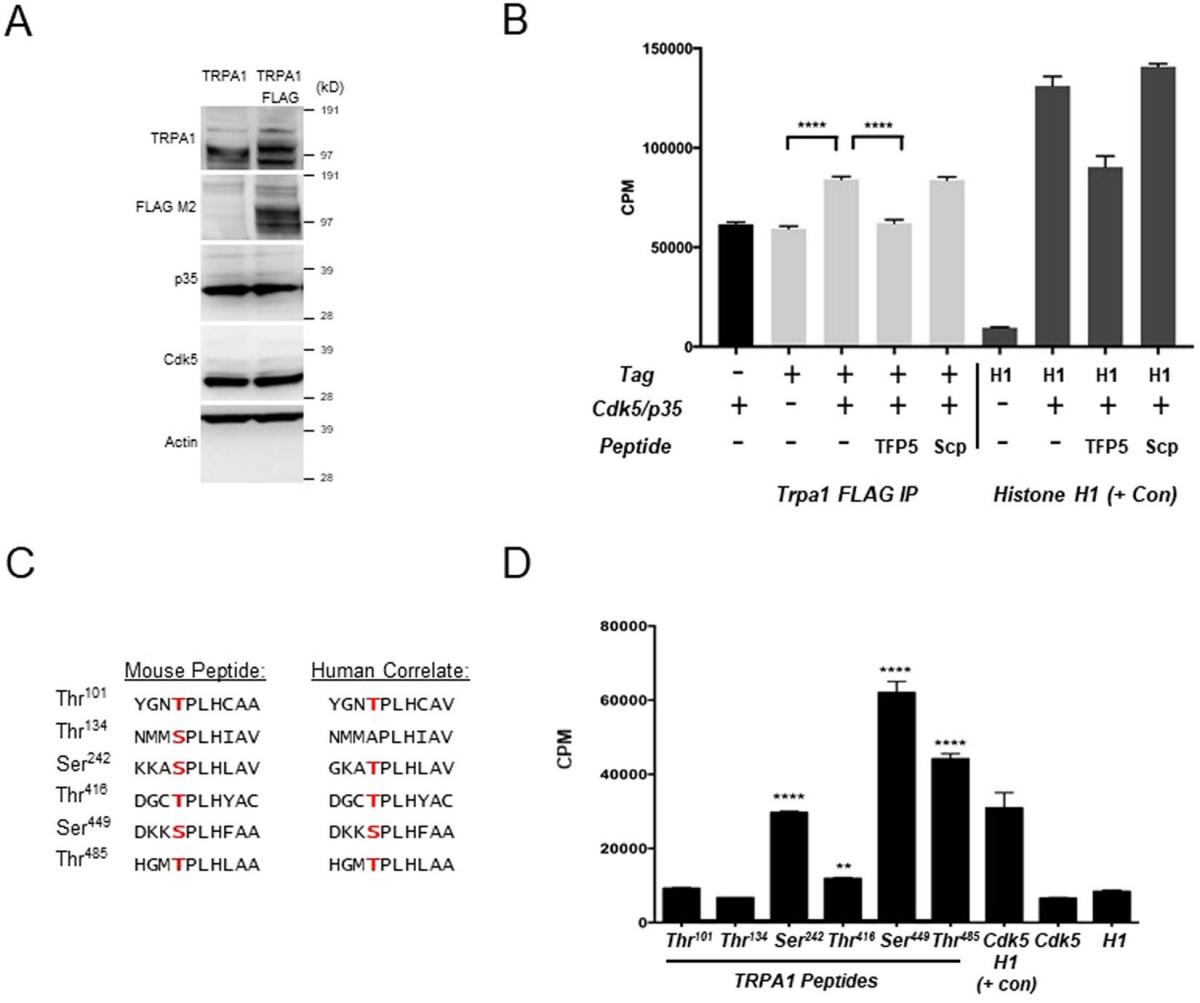
Cdk5 phosphorylates sites in the TRPA1 protein. (**A**) A FLAG-tagged version of TRPA1 was expressed in Neuro-2a cells along with an untagged TRPA1. (**B**) Immunoprecipitated FLAG tagged TRPA1 was phosphorylated by recombinant Cdk5/p35. The tagged TRPA1 shows higher ^32^P incorporation as compared to the results without kinase (one-way ANOVA, ****P < 0.0001). Untagged TRPA1 was also used as an immunoprecipitation control against the FLAG tag. The Cdk5 inhibitory peptide TFP5 was able to block phosphorylation of the tagged TRPA1, demonstrating that the addition of ^32^P was specifically mediated by Cdk5 activity (one-way ANOVA, ****P < 0.0001). In contrast, a scrambled peptide (Scp) had no effect on kinase activity. Histone H1 was used as positive control to show Cdk5/p35 kinase activity. (**C**) Sequence of mouse TRPA1 peptides used for the Cdk5 *in vitro* kinase assay. Corresponding human TRPA1 sequences also shown. (**D**) Synthetic TRPA1 peptides based on the putative Cdk5 motifs in the mouse TRPA1 were incubated with recombinant Cdk5/p35 while kinase activity was measured using ^32^P incorporation. Histone H1, a known substrate of Cdk5, was used as positive control when incubated with recombinant Cdk5/p35. Results were compared to either Cdk5/p35 or histone alone, both of which were used as negative controls (unpaired *t*-test, **P < 0.01 & ****P < 0.0001).

In order to determine if TRPA1 is a substrate of Cdk5, we immunoprecipitated a full length FLAG-tagged TRPA1 and tested it in an *in vitro* kinase assay using recombinant Cdk5/p35. Neuro-2a cells were transfected with an expression vector for TRPA1 that included a C-terminal FLAG tag (Fig. 2C). The FLAG-tagged TRPA1 was then immunoprecipitated using anti-FLAG affinity beads and subsequently incubated with recombinant Cdk5/p35. An untagged version of TRPA1 was used as an immunoprecipitation background control. FLAG-tagged TRPA1 showed significantly higher ^32^P incorporation in the presence of Cdk5/p35 than baseline levels without inclusion of the active recombinant kinase (Fig. 2D). A Cdk5 inhibitory peptide, TFP5, was also used to demonstrate that the ^32^P incorporation was dependent on Cdk5 activity^35^, whereas a scrambled peptide had no effect on the kinase assay.

After determining that TRPA1 was a substrate of Cdk5, the next objective was to determine which of the six potential Cdk5 sites in TRPA1 were most amenable to phosphorylation. We, therefore, conducted an *in vitro* Cdk5 kinase assay employing 10 residue amino acid peptide substrates based on each of the 6 potential sites and centered on the obligate proline residue (Fig. 2A). We had previously employed a similar peptide-based approach to locate the most probable of 3 potential Cdk5 sites in TRPV1^6^. The TRPA1 peptides were incubated with recombinant Cdk5/p35 and tested for ^32^P incorporation. Two potential sites, T^101^ and T^134^, did not show ^32^P incorporation levels above background (Fig. 2B). In contrast, S^449^ and T^485^ are highly phosphorylated by Cdk5 *in vitro* (Fig. 2B). Both the S^449^ and T^485^ flank a reported EF-hand like domain^41^ that may be involved in local structural perturbation, but does not appear to play a role in calcium gating of TRPA1^44^. Rather, it appears that interactions with calmodulin at the TRPA1 C-terminal predominantly determines Ca^2+^-dependent potentiation and inactivation^45^. These residues and the surrounding amino acids of the motif are also highly conserved in vertebrates and invertebrates (Supplementary Fig. 3B). Another Cdk5 site significantly phosphorylated is S^242^ (Fig. 2B), which is found in AR6, a region suggested to be involved in thermal activation^46^. T^416^ is marginally phosphorylated, but interestingly is flanked by two reactive cysteines residues (C^415^ and C^422^)^34^.

### Neuronal Responses to the TRPA1 Agonist, AITC, are Regulated by p35 Levels

**Figure 3:**
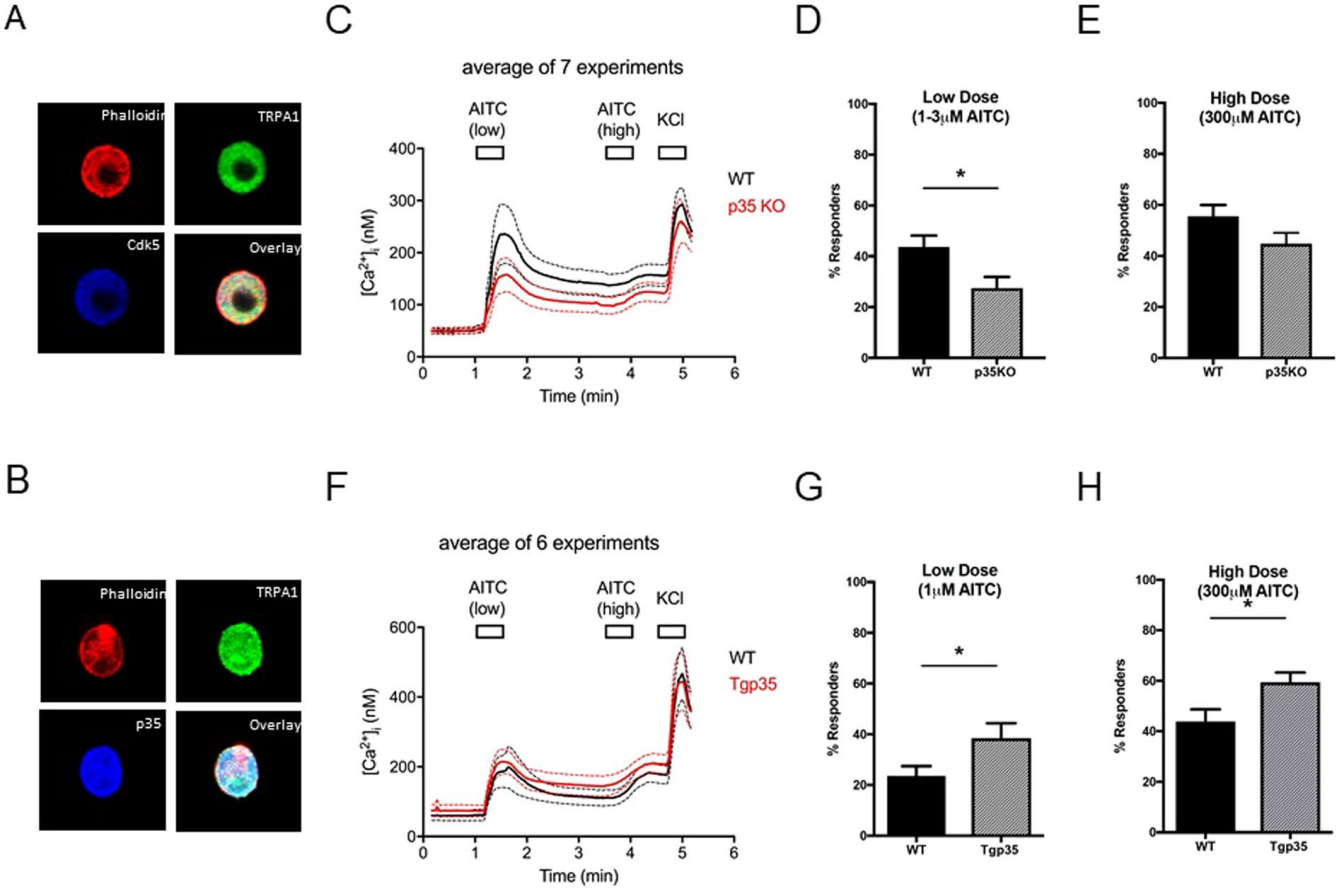
Calcium imaging of trigeminal neurons after stimulation with allyl isothiocyanate (AITC). (**A** and **B**) Both Cdk5 and its activator p35 are co-expressed with TRPA1 in cultured trigeminal ganglia neurons. Ca2^+^ imaging was performed using cultured trigeminal neurons from mice genetically engineered to have either increased (Tgp35) or decreased (p35KO) Cdk5 activity. Phalloidin, which binds to F-actin, was used as a control to stain individual cells. Cultured neurons were treated with either a low (1–3 μM) or high (300 μM) concentration of the TRPA1 agonist AITC. The percent of responders was normalized to KCl. (**C**) Cultured neurons from the p35KO mice (n = 7). Averaged Ca^2+^ imaging traces ± SEM from 7 experiments using p35KO mice and littermate controls. (**D**,**E**) The p35KO mice show fewer % responders at a low dose of the TRPA1 agonist AITC as compared to the wild type mice (unpaired *t*-test, *P < 0.05), but no significant differences were seen at high doses of AITC. (**F**) Averaged Ca^2+^ imaging traces ± SEM from 6 Tgp35 mice and wild type controls. (**G**,**H**) In contrast to the p35KO mice, cultured neurons from Tgp35 mice (n = 6) tend to be more responsive to AITC at both low and high concentrations.

After identifying potential sites favorable for phosphorylation by Cdk5 within the structure of TRPA1, we determined whether Cdk5 activity had any effect on TRPA1-mediated Ca^2+^ influx in cultured trigeminal ganglia (TG) neurons. We first verified the co-expression of TRPA1 with Cdk5 and p35 in cultured neurons using immunofluorescence. In Fig. 3A, Cdk5 and TRPA1 are co-expressed in dissociated cultured trigeminal ganglia neurons. Next, we performed immunofluorescence for p35, a regulatory subunit that binds to Cdk5 via a “cyclin box” to modulate kinase activity. Levels of p35 are also increased during inflammation, which, in turn, activates Cdk5 to produce increased pain sensitivity^47^. Like Cdk5, p35 is co-expressed with TRPA1 in dissociated TG sensory neurons as well (Fig. 3B). Along with immunofluorescence, our analysis of available single cell RNA-sequencing databases from mouse DRG neurons shows that 100% of TRPA1^+^ neurons express Cdk5 (Supplementary Figure 1). The t-distributed stochastic neighbor embedding (t-SNE) plots in Supplementary Figure 4C additionally show that all TRPA1^+^ neurons coexpress Cdk5, but, conversely, not all Cdk5^+^ neurons express TRPA1, as Cdk5 is uniformly expressed amongst all DRG neuronal subtypes.

With confirmation that Cdk5 and its activator p35 are both expressed in TRPA1^+^ neurons, we used Ca^2+^ imaging to measure changes in cytosolic Ca^2+^ [Ca^2+^_i_] caused by TRPA1-dependent Ca^2+^ entry in cultured dissociated TG neurons derived from mice that were genetically engineered to have either increased or decreased Cdk5 activity. We turned to genetically engineered mice with altered expression of the Cdk5 activator p35, which acts as a rate-limiting determinant of kinase activity. We prepared cultured primary neurons from either p35KO mice (p35 knockouts with >85% reduction in Cdk5 activity) or Tgp35 mice (a transgenic mouse line overexpressing p35 that has a >50% increase in Cdk5 activity)^1,2^. To determine the extent of TRPA1 activation in dissociated neurons, we used allyl isothiocyanate (AITC), a pungent plant compound found in mustard oil. AITC is an electrophilic agonist of TRPA1 that covalently binds to the reactive cysteines Cys^415^, Cys^422^, and Cys^622^ in the mouse TRPA1^34^. In particular, Cys^415^ (Cys^414^ in humans) is thought to be important for channel activation with both cysteine and non-cysteine reactive TRPA1 agonists^48^ and this electrophilic site is intriguingly situated adjacent to the potential Cdk5 phosphorylation site T^416^. Cultured trigeminal neurons from p35KO and Tgp35 mice were loaded with Fura 2-AM, tested with a low concentration of AITC, washed, bathed in a high concentration of AITC, washed, then treated with 30 mM KCl to determine the total excitable neuronal component of the culture (Fig. 3C,F).

Initially, we compared TRPA1 influx at a low dose of 3 μM of AITC, followed by a saturating dose of 300 μM. We subsequently examined TRPA1 activity at a lower concentration of 1 μM with similar results. When dissociated TG neurons obtained from WT mice were exposed to AITC, a subpopulation of neurons showed an increase in intracellular Ca^2+^ levels. 1 or 3 μM AITC produced only modest responses, whereas 300 μM AITC produced a greater Ca^2+^ response (Supplementary Figure 5A). In many neurons, however, 1 or 3 μM AITC evoked a large response during a 30 sec application, which was only partially recovered toward baseline following washout. In such cases, 300 μM AITC did not evoke any response, which is presumably due to desensitization of TRPA1. When the amplitude of response was calculated by fold increase from baseline level, the response evoked by the low concentration of AITC was 1.59 ± 0.14 fold of baseline (n = 7 mice). Since response to 300 μM was apparently affected by preceding response to low dose AITC, we did not calculate the amplitude of response at the higher concentration. When we applied a cutoff of 1.15 fold, 43.6 ± 4.5% neurons were estimated to be responders (n = 7 mice). When dissociated TG neurons obtained from p35 KO mice were exposed to AITC, the amplitude of response (1.38 ± 0.08; n = 7 mice) was substantially smaller than that of WT but they were not significantly different (Fig. 3C). However, we consistently observed decreased numbers of responding neurons at low concentrations of AITC from cultures derived from the p35KO mice versus the controls (27.4 ± 4.4% in p35KO vs. 43.6 ± 4.5% in WT; n = 7 mice in each group; P < 0.05, unpaired t-test) (Fig. 3D,E). These results were contrasted with Ca^2+^ imaging using dissociated TG neurons obtained from Tgp35 mice. These transgenic mice overexpress p35 under the control of the p35 promoter to induce increased neuronal Cdk5 activity^48^. In comparison to the p35 knockouts, the TG neurons from the Tgp35 mice exhibit slightly greater responses to the low concentration of AITC than WT (1.30 ± 0.07 fold in WT; 1.37 ± 0.04 fold in Tgp35; n = 6 mice in each group) but the difference was not statistically significant (Fig. 3F). When the proportion of responders were compared, TG neurons from Tgp35 mice showed increased percentages of responders at both low and high concentrations of AITC (38.4 ± 6.0% in Tgp35; 23.5 ± 3.9% in WT at 1 μM and 59.4 ± 3.9% in Tgp35 vs. 43.8 ± 5.0% in WT at 300 μM, n = 6 mice in each group; P ≤ 0.05, unpaired t-test) (Fig. 3G,H). It is notable that the proportion of responders was much lower in littermate FVB/N WT for Tgp35 than that in littermate C57BL/6 WT for p35KO. Although the source of difference is not clear, we presume it is due to the different background strains.

### Cdk5 Activity Regulates Aversion to Oral AITC in Mice

**Figure 4:**
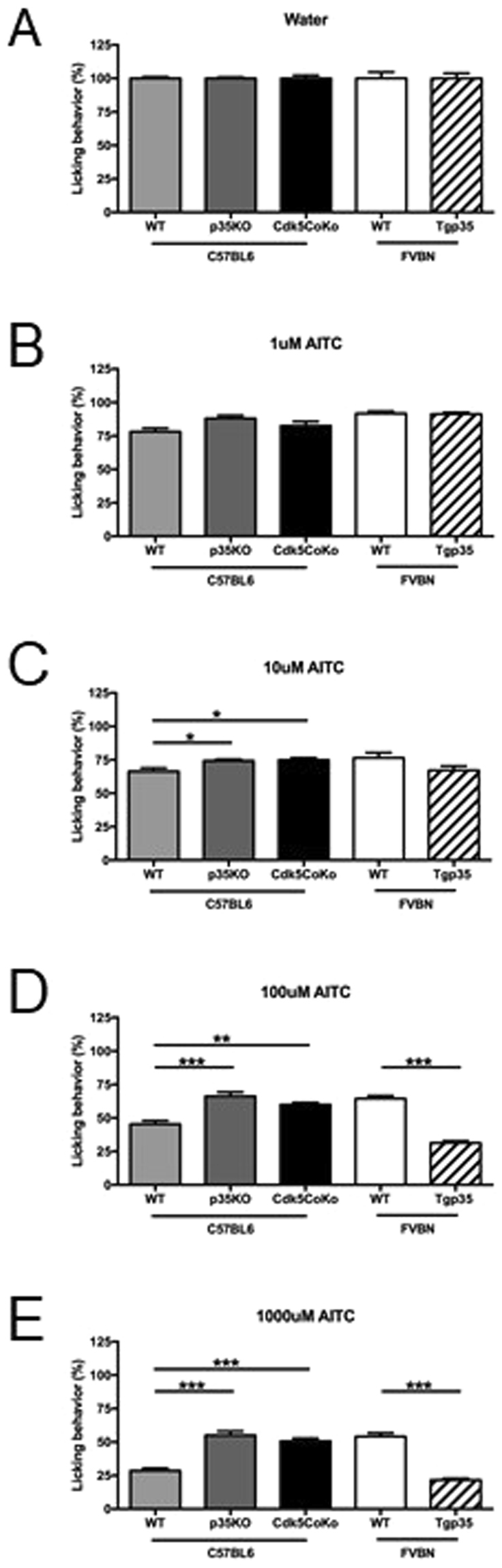
Response to allyl isothiocyanate (AITC) in animals with genetically driven alterations in Cdk5 kinase activity. (**A**–**E**) Genetically engineered mice with either increased or reduced Cdk5 activity were tested for oral aversion to AITC using a lickometer. Water-deprived C57BL/6 and FVB/N mice were tested for 1 h using the lickometer with a free access to water containing different concentrations of AITC. The behavior is expressed as a % of the baseline licking responses for plain water as compared to AITC. The p35KO mice and Cdk5CoKo mice (C57BL/6:129/SvJ background) were less sensitive to water dosed with AITC (increased licking behavior) as compared to the controls (one way ANOVA, *P < 0.05 at 10 μM, *P < 0.051 at 100 μM and ***P < 0.001 at 100 & 1000 μM). Conversely, the Tgp35 mice show higher aversion and hypersensitivity to 100 and 1000 μM AITC (unpaired *t*-test, ***P < 0.001) as evident by the decreased number of licks. SEM from four animals during five different measurements.

The Ca^2+^ imaging experiments determined that Cdk5 activity influences the responsiveness of neurons to treatment with AITC. We next wanted to determine if the differences seen in the cultured neurons would translate into altered behavioral responses to AITC *in vivo*. TRPA1 is essentially co-localized along with TRPV1 in the nerve terminals that innervate the oral mucosa and nasal cavity, where it acts to detect diverse pungent plant compounds like those within mustard oil, cinnamon, and garlic^49^. To examine oral chemo-nociceptive aversion to the pungent TRPA1 agonist AITC, genetically-engineered mice with either increased or decreased Cdk5 activity were tested using device known as the lickometer. Measurement of the licking behavior of our mice was conducted in response to ascending concentrations of AITC (1, 10, 100, and 1000 μM) in the drinking water. Mice were first habituated to the lickometer with water only prior to testing with AITC. During the training sessions, we observed no change in the number of licks between the different genotypes of mice and their corresponding controls (Fig. 4A). The lowest dose of AITC revealed no differences in the licking responses within the genotypes (Fig. 4B). At higher AITC concentrations, the p35KO mice showed significantly less aversion to AITC-containing water than wildtype mice (P ≤ 0.05 at 10 μM, P ≤ 0.001 for 100 μM and 1000 μM) (Fig. 4C–E). Conversely, the Tgp35 mice exhibited increased aversion to the higher concentrations of AITC (P ≤ 0.001 for both 100 μM and 1000 μM). These results seen with our two Cdk5 mouse models parallel those obtained with the sensory neurons *in vitro* and support the idea that Cdk5 activity modulates the responses transduced *in vivo* via TRPA1 channel activation.

For lickometer testing, we need to account for the fact that both p35KO and Tgp35 mice are engineered with a global genetic mutation throughout the mouse, including the central nervous system. We, therefore, wanted to limit disruption in Cdk5 activity only to the nociceptive neurons by using a C-fiber specific Cre (SNS-Cre) to conditionally knockout Cdk5 (Cdk5CoKo)^6^. We tested oral aversion to AITC in the Cdk5CoKo mice that lack Cdk5 activity in small-diameter neurons of the TG, which are known to relay orofacial nociception. Cdk5CoKo mice were tested for oral aversion to AITC and showed similar responses to p35KO mice, as shown by increased licking versus the controls (P ≤ 0.05 at 1 μM, P ≤ 0.01 for 100 μM, and P ≤ 0.001 at 1000 μM, Fig. 4D,E).

## Discussion

Upregulated Cdk5 activity within primary sensory neurons modulates pain sensitivity by directly adjusting the responses of these neurons to painful stimuli^1,2,6^. We further investigated the extent to which Cdk5 phosphorylation could be a multifactorial regulator of nociceptive inputs by trying to identify new Cdk5 substrates that are highly expressed in nociceptive afferents, particularly the TRPV1^+^ set of ganglionic neurons which are critical for the detection of many painful modalities, especially heat^50^. Within the general population of nociceptive neurons, we identified multiple candidate proteins that contain putative Cdk5 phosphorylation sites, which could then affect the excitability of these neurons, and thereby modulate the nociceptive tone. Out of this group, we focused on TRPA1 because further examination revealed six potential sites that were in the same general location within the N-terminal ankyrin repeat-containing domains. TRPV1 is also regulated by Cdk5^6^ and our analyses of the single cell DRG data sets show approximately 50% coexpression, further supporting a role for Cdk5 phosphorylation in regulating these two key transducers of noxious stimuli in multiple subpopulations of afferent nerve terminals.

TRPA1 is a pain-transducing ion channel that is primarily known as a chemo-sensor in nociceptive neurons, but is also implicated in cold and mechanical hypersensitivity^19,51^. TRP channels, in general, are known to be modulated by phospholipase C (PLC), as the sensitization of TRPA1 following stimulation of either PAR2 or the bradykinin receptor 2 (B_2_R) is mediated by PLC activity^23,25^. TRPA1 channel activity induced by bradykinin is inhibited by protein kinase A inhibitors, and activation of either PKA or PLC leads to membrane trafficking of TRPA1^52^. Recently, TRPA1 was shown to be phosphorylated by PKA at the amino acid residues S^86^, S^317^, S^428^, and S^972^, which, in turn, has a sensitizing effect on TRPA1^27^. Brackley *et al*. concurrently demonstrated PKA-mediated phosphorylation of TRPA1, with the mouse S^87^ residue being key to sensitizing mustard oil responses. In addition, Brackley *et al*. showed phosphorylation of TRPA1 by protein kinase C (PKC) as well, where the S^119^, T^281^, and T^529^ PKC sites are essential for sensitization^28^. During both PKA and PKC-mediated phosphorylation of TRPA1, the scaffolding protien AKAP (A-Kinase Anchoring Protein 79/150) plays an essential role in anchoring these kinases to TRPA1.

During our bioinformatics search for Cdk5 substrates associated with pain signaling, we observed that TRPA1 has many putative Cdk5 sites within its ankyrin repeats, which could potentially modify channel opening or conductance and, ultimately, modulate the efficacy of signal transduction through this channel. The six potential Cdk5 phosphorylation sites in TRPA1 are all located in the ankyrin repeats that contain a canonical pattern of [G-(X)-TPLH-(X)-A-(X3)-G-(X7)-LL-(X2)-GA-(X5)]^31^. Structural modeling revealed a stereotypical pocket within these ankyrin repeats that can accept addition of the phosphate group. Using *in vitro* kinase assays, we demonstrated that recombinant full-length TRPA1 is a substrate for Cdk5, and identified which putative potential Cdk5 sites could act as preferred substrates for phosphorylation. These sites are highly conserved in both mammals and lower organisms such as mosquitos and fruit flies where TRPA1 serves as a thermosensor^53,54^.

In our report, we wanted to examine Ca^2+^ imaging on dissociated cultured AITC-responsive nociceptors from our mouse models with genetically altered levels of p35, but with endogenous expression of TRPA1 and Cdk5. We observed that primary neurons from p35 knockout mice, which lack the required cofactor for Cdk5 activity, exhibited a reduced percentage of responders to AITC (Fig. 3C,D). Conversely, overexpression of p35 enhanced the percentage of responders to either a low or a high dose of AITC (Fig. 3E,F). These data support our hypothesis that Cdk5 regulates the activity of TRPA1. However, Ca^2+^ imaging in sensory neurons does not directly test the activity of TRPA1 and likely involves multiple TRPA1-dependent (see Supplementary Table 1) and potentially TRPA1-independent factors. For example, Cdk5-mediated regulation of other molecules such as both high voltage-activated P/Q- and N-type Ca_V_ channels and low voltage-activated T-type channels  can also modulate neuronal excitability^55^. Therefore, more direct measurement of TRPA1 function should help to further support our hypothesis. Using our genetically engineered mouse models, we expanded upon the Ca^2+^ imaging to study behavioral avoidance responses to AITC, where we demonstrated that Cdk5 activity influences aversion to AITC *in vivo* in a concentration dependent manner (Fig. 4). These experiments demonstrate that Cdk5 activity affects responses to the TRPA1 agonist AITC both in terms of Ca^2+^ influx in cultured primary TG neurons and with the lickometer to measure aversive behavior.

During the course of our study, Hynkova *et al*.^56^ demonstrated that cotransfection of human TRPA1 and p35 in HEK293T cells resulted in increased electrophysiological responses to cinnamaldehyde, a TRPA1 agonist. While in agreement with our findings, they ascribe Cdk5′s effects on TRPA1 to phosphorylation of T^673^, even though the typical (T/S)PLH consensus motif within the ankyrin repeats of TRPA1 were predicted to be Cdk5 phosphorylation sites at a high stringency level. The amino acid analogous to the human TRPA1 T^673^, however, is an alanine (A^675^) in the mouse TRPA1 (Supplementary Figure 3C), essentially resulting in a phospho-null version of TRPA1, but our results from cultured TG neurons and the lickometer behavioral testing suggest that Cdk5 activity still retains the ability to modulate TRPA1 responses in mice. In addition, immunoprecipitated FLAG-tagged mouse TRPA1 can still be phosphorylated by Cdk5 even with this amino acid substitution, and our *in vitro* kinase assay shows that the (T/S)PLH motif common to many ankyrin repeats can be highly phosphorylated by Cdk5. Our *in vitro* kinase data suggests that these tetrapeptide motifs are still the most likely Cdk5 phosphorylation sites, but further analysis needs to be conducted to determine which ankyrin repeat phosphorylation site is most critical for sensitizing TRPA1.

Various techniques to assess the number of TRPA1^+^ neurons in the DRG suggest a range between 3.6% to 32.4% in rodents^14,20^. TRPA1 expression, however, can also be increased following tissue injury^20^. Taking advantage of the two available single cell transcriptomic murine data sets^57,58^ a comparative analysis suggests that 43% of DRG neurons express TRPA1 in the mouse. Lennertz *et al*.^51^ suggest that there may be a nociceptor population of C-Mechano Cold (CMC) fibers involved in mechanical hyperalgesia that express TPRA1 only, while inflammatory heat hypersensitivity occurs via a separate population of neurons expressing TRPV1. Other studies, however, show that TRPA1^+^ neurons predominantly co-express TRPV1^14,59^. Our transcriptome analysis shows that TRPA1 is highly enriched in TRPV1^+^ neurons^10^. Co-expression of both of these TRP channels in nociceptive neurons is additionally supported by examples of cross-desensitization between TRPV1 and TRPA1 agonists, as capsaicin pretreatment in rats reduces subsequent AITC responses and vice versa^60^. Bautista *et al*.^61^ also proposed that TRPV1-mediated calcium influx activates TRPA1 downstream of bradykinin signaling, which is known to promote TRPA1 sensitization. Weng *et al*.^62^ showed that Tmem100 links TRPV1 and TRPA1 in nociceptive neurons, which then promotes TRPA1 Ca^2+^ influx by weakening the association between these two channels to promote TRPA1 activity. Conditional deletion of Tmem100 was, therefore, able to reduce inflammatory-induced mechanical hypersensitivity but did not alter heat responses. In contrast to Tmem100-related trafficking of these two TRP channels, TNF-α signaling was also shown to promote plasma membrane localization of both TRPA1 and TRPV1, mediated by Munc18-1/syntaxin1/Snap-25 complexes^63^. Interestingly, TNF-α signaling is known to induce p35 expression, which, in turn, increases Cdk5 activity^47^. The resulting active Cdk5/p35 complex could then, in turn, phosphorylate both Munc18^64^ to regulate exocytosis, and the ion channels TRPV1^6^ and TRPA1, which subsequently encourages Ca^2+^ influx and depolarization. With TRPA1 primarily localized within many TRPV1^+^ neurons^14^ and both of these pain-transducing ion channels acting as Cdk5 substrates, we decided to develop a gene co-expression map of putative Cdk5 substrates using single cell RNA-sequencing data from mouse dorsal root ganglia (Supplementary Figure 4). The gene co-expression map hints at a likely complex interplay between TRPV1, TRPA1, and the active p35-bound Cdk5 kinase within nociceptive neurons.

New therapeutics to treat pathological pain are needed, particularly since pain accounts for 20% of doctor visits, and 10% of drug sales are for treating pain^65^. Attempts to pharmacologically inhibit either TRPV1 or TRPA1, however, have been unsuccessful^66^ or are still in progress. TRPV1 antagonists cause hyperthermia while also lessening protective responses to noxious heat. TRPA1 antagonists, on the other hand, have not moved past Phase II clinical trials. Protein kinases have become intriguing new pharmacological targets to treat pain because of their involvement with peripheral and central sensitization^67^. Along with TRPV1, we have now shown that Cdk5 can also phosphorylate the pain-transducing ion channel TRPA1 and modulate its activity, which suggests that Cdk5 activity plays an important role in modifying pain sensitivity within nociceptive neurons, particularly following inflammation. Targeting Cdk5, therefore, provides an ideal means of inhibiting both TRPA1 and TRPV1 hypersensitivity, which are associated with mechanical and heat hyperalgesia, respectively. Lastly, inhibiting a protein kinase like Cdk5 would hopefully block pathological pain responses related to primary sensitization without blocking normal physiological nociception.

## Methods

### Bioinformatics search

Amino acid preference of Cdk5 was probed using a position-specific scoring matrix originally developed by Borquez *et al*.^29^ and Scansite 2.0^68^, which has a built-in search sequence for Cdk5 phosphorylation sites. Results from this screen were filtered by selecting for highly expressed genes in the dorsal root ganglia (DRG) TRPV1-expressing lineage as determined by previously published RNA-Seq datasets of this tissue^10,11^ (Supplementary Table 1). Previously annotated phosphorylation sites were reviewed using PhosphoSitePlus^69^. Alignments were performed using MultAlin^70^.

### Structural Modeling

Atomic coordinates of proteins determined using structural data from the Brookhaven Protein Databank (PDB)^71^. The following TRP channel structural models, including their complexes with ligands given by their PDB designation used were: 2nyj 3j5p 3j5q 2eta 3j9p. Protein sequence of the human TRPA1 was retrieved from GenBank^TM^ (GenBank NM_177781.4). The theoretical model of TRPA1 was obtained from The School of Theoretical Modeling where it was generated using methods described previously^72,73^. The homology with ankyrin repeats of TRPV1 and TRPV2 was used as an aid in the contrstruction of our model of AR1 to 11 because of similarities to the repeats in TRPA1 (See Supplementary Fig. 3A for comparisons of the TRPA1 ankyrin repeats to AR3-6 of TRPV1). The region of amino acid similarity between TRPA1 and TRPV1 is also in the region of ATP and PIP2 binding as well as regulation by tachyphylaxis in TRPV1. Similarities of TRPA1 amino acid sequence to that of TRPV1 and TRPV2 in only this selected region suggest similar properties for multiligand binding of factors such as soluble N-ethylmaleimide sensitive factor SNARE and acyl-coA binding domain protein 3 for TRPV1 and TRPA1, respectively. The phospho-group at T^485^ was modeled in the crystal structure of TRPA1 (pdb code 3j9p) and in a predicted model of the ankyrin repeat domain (theoretical model code trpa).

### Cdk5 Kinase Assay

Recombinant full-length human Cdk5 and p35 (Sigma-Aldrich, St. Louis, MO) were incubated in 45 μl of kinase assay buffer (100 mM Tris·HCl [pH 7.4]/50 mM MgCl_2_/5 mM EDTA/50 μM NaF/5 μM Na_2_VO_3_/5 mM DTT) containing either 10 μg of histone H1 (Sigma-Aldrich), immunoprecipitated TRPA1, or the following mouse TRPA1 peptides (Peptide 2.0, Chantilly, VA): TRPA1^1^ YGNTPLHCAA, TRPA1^2^ NMMSPLHIAV, TRPA1^3^ KKASPLHLAV, TRPA1^4^ DGCTPLHYAC, TRPA1^5^ DKKSPLHFAA, TRPA1^6^ HGMTPLHLAA. Kinase assays were carried out at 30 °C for 60 min by adding 5 μCi of [γ-^32^P] ATP (0.5 mM). The peptide assay was stopped by adding 10% trichloroacetic acid to precipitate the proteins. 20-μl aliquots of trichloroacetic acid supernatant were transferred onto P81 phosphocellulose squares (spotted in duplicates), air-dried, and washed five times for 15 min each in 75 mM phosphoric acid and once in 95% ethanol. After air drying, squares were transferred to vials containing Bio-Safe II scintillation fluid (Research Products International, Mount Prospect, IL) for counting in a Beckman Coulter (Fullerton, CA) scintillation counter (model no. SL 3801).

### Tissue Culture and Immunoprecipitation

FLAG tagged and untagged mouse TRPA1 expression vectors were purchased from Origene (Rockville, MD). The mouse neuroblastoma cell line Neuro-2a was cultured in DMEM and 10% fetal bovine serum and the cells were transfected with the TRPA1 constructs using Neuro-2a Cell Avalanche Transfection Reagent (EZ Biosystems, College Park, MD). Western blot was performed as previously described^20^. TRPA1 expression was confirmed using the following antibodies: FLAG M2 (Sigma-Aldrich), TRPA1 (Wako Chemicals, Richmond, VA), p35 (Santa Cruz, Santa Cruz, CA), Cdk5 (PhosphoSolutions, Aurora, CO), and Actin (Millipore, Billerica, MA). Immunoprecipitation of FLAG tagged TRPA1 was performed using ANTI-FLAG M2 Magnetic Beads (Sigma-Aldrich), according to the manufacturer’s protocol.

### Immunofluorescent Staining of TG Neurons

Trigeminal ganglia (TG) neurons were plated onto Lab-Tek 8 chamber cover-slides (Thermo Fisher Scientific, Waltham, CA) coated with laminin/poly-D-lysine (Sigma-Aldrich) with F12 medium (Thermo Fisher Scientific) containing 10% FBS (HyClone Laboratories, Logan, UT) and 1% penicillin/streptomycin (Thermo Fisher Scientific). The following day, cultured cells were fixed in 4% paraformaldehyde (Electron Microscopy Sciences, Hatfield, PA) and permeabilized for 10 minutes with 0.1% Triton X-100 (Sigma-Aldrich). Cells were probed with the following primary antibodies: TRPA1 (Novus Biologicals LLC, Littleton, CO), p35 (C-19) and Cdk5 (C-8) (both Santa Cruz Biotechnology). After several washes with PBS (Thermo Fisher Scientific), cultured neurons were incubated with secondary antibodies conjugated with either Alexa 488 or Alexa 647 (1:500, Molecular Probes, Carlsbad, CA) and finally labeled with rhodamine-phalloidin (1:300, Molecular Probes, Carlsbad, CA) for 20 minutes. All the images were acquired using a Zeiss LSM 700 microscope.

### Animals

p35 knockout (p35KO) and Cdk5 conditional knockout (Cdk5CoKo) mice were maintained in a C57BL/6:129/SvJ background. Transgenic p35 (Tgp35) mice were conserved in an FVB/N background. Age-matched wild-type mice served as controls. C-fiber neuron-specific Cdk5CoKo mice were generated as previously described^6^ where an SNS (NaV1.8) promoter-driven Cre is used to conditionally delete Cdk5 in small-diameter nociceptive neurons. All animals were housed in standard cages in climate- and light-controlled rooms with free access to food and water. All experimental procedures were approved by the Animal Care and Use Committee of the National Institute of Dental and Craniofacial Research, National Institutes of Health and we adhered to the guidelines of the IASP Committee for Research and Ethical Issue^74^.

### Dissociation of TG neurons and Ratiometric Ca^2+^ imaging

Ca^2+^ imaging was performed as previously described^75^. Essentially, the trigeminal ganglia (TG) were extracted from p35KO and Tgp35 mice and their respective wild type (WT) littermate controls. Minced TG were incubated in Ca^2+^-free, Mg^2+^-free HBSS with 1 mg/ml collagenase type IV (Sigma-Aldrich, St. Louis, MO) at 37 °C for 30 min with agitation. EDTA, DNAse, and 2.5% trypsin were then added and the tissues were incubated for an additional 15 min at 37 °C. Following enzymatic digestion, TG neurons were centrifuged, resuspended in culture medium, and mechanically dissociated using a descending diameter firepolished glass micropipettes. Dissociated neurons were plated onto glass coverslips (8-mm) coated with poly-ornithine and laminin with DMEM/F12 medium containing 10% FBS and 1% penicillin/streptomycin. The following day, cultured neurons were loaded with Fura2-AM and pluronic acid for 45 minutes at 37 °C in calcium imaging buffer (CIB; NaCl 130, KCl 3, 0.5 MgCl_2_, CaCl_2_ 0.9, HEPES 10, sucrose 10, NaHCO_3_ 1.2 [in mM] [pH 7.45, 320 mOsm adjusted with mannitol]). Ratiometric Ca^2+^ imaging was performed using an inverted fluorescence microscope (Nikon Instrument, Melville, NY), an excitation filter changer (Sutter Instrument, Novato, CA), and a CCD camera (Nikon Instrument). Paired images (340- and 380-nm excitation, 510-nm emission) were collected every 2 s with NIS Element (Nikon Instrument). The Fura response was defined as the ratio of emissions measured during excitation at 340 and 380 nm, and the relative Fura response was defined as the ratio normalized to the five ratio values prior to the application of drug. Conversion of Fura ratios to absolute [Ca^2+^]_i_ was performed following system calibration^76^ using a calcium calibration kit (Thermo Fisher Scientific, C3008MP). The amplitude of response was calculated by fold increase from baseline level. The response level was defined as the level 3 standard deviations above the mean of the nonresponsive peak, which was 1.15 times (15% increase) greater than their average baseline response prior to the application of AITC. The percentage of responders was calculated by normalizing the number of responders to the number of KCl responders in entire neurons derived from one mouse. AITC-evoked Ca^2+^ responses were various in different experiments. To control for the effects of different experimental conditions, we tested a pair of transgenic mouse and a littermate WT in the same day. The averaged response amplitudes and percentage of responders were obtained from each experiment and data from 6 to 7 mice were compared. In the p35 KO mice, a total of 217 neurons from 7 mice (31 ± 7.0 neurons per mouse) were analyzed. In the corresponding C57BL/6 littermate WT controls, a total 238 neurons from 7 mice (34 ± 9.7 neurons per mouse) were analyzed. In Tgp35 mice, a total 231 neurons from 6 mice (34 ± 8.4 neurons per mouse) were analyzed. In the corresponding littermate FVB/N WT mice, a total 204 neurons from 6 mice (38.5 ± 6.1 neurons per mouse) were analyzed.

### Mouse Operant Lickometer Test

An operant lickometer test was used to test nociceptive responses to noxious oral stimuli. Nociceptive sensitization was induced by different concentrations of the TRPA1 agonist AITC (Sigma-Aldrich, St. Louis, MO). Water-deprived (15 h) animals were placed in the lickometer cages (Habitest system, Coulbourn Instruments, Whitehall, PA) where a computer-operated system monitored their licking behavior for a period of 1 hour. Initially, the animals were tested with water (N = 5 sessions). Afterwards, consumption/aversion to water with different concentrations of AITC was monitored (N = 5 sessions per single AITC concentration). All mice were tested at the same time each day and then retested under the same conditions on non-consecutive days.

### Statistical analysis

All data are expressed as mean ± SEM. The statistical evaluation was done with GraphPad Prism 7 software (GraphPad, San Diego, CA, USA). Statistical differences between the groups were assessed using either ANOVA or an unpaired t-test with significance level was set at p < 0.05.

### Cdk5 Substrate Network Analysis

The gene coexpression network was built using publicly available RNA-sequencing data of mouse dorsal root ganglion^57,58^ (SRA database, PRJNA268295). The threshold for positive gene expression was 5 FPKM (fragments per kilobase of transcript per 10^6^ mapped reads). Network visualization was constructed with R software using package ‘igraph’^77^. RNA-sequencing data from whole tissue rat DRG^2^ (SRA database, PRJNA308243) was used to quantitate DRG expression levels for putative Cdk5 substrates.

## Electronic supplementary material

Supplementary Information
