# Supplementary material for: Phosphorylation of the Transient Receptor Potential Ankyrin 1 by Cyclin-dependent Kinase 5 affects Chemo-nociception
Source: Sci Rep. 2018 Jan 19;8:1177. doi: 10.1038/s41598-018-19532-6 (PMC5775258; doi:10.1038/s41598-018-19532-6)

Supplementary Material

Phosphorylation of the Transient Receptor Potential Ankyrin 1 by Cyclin-dependent Kinase 5 affects Chemo-nociception

Bradford E. Hall[1, *], Michaela Prochazkova[1, *], Matthew R. Sapio[2], Paul Minetos[1,#], Natalya Kurochkina[3], Binukumar BK[4], Niranjana D. Amin[5], Anita Terse[1], John Joseph[6], Stephen J. Raithel[2], Andrew J. Mannes[2], Harish C. Pant[5], Man-Kyo Chung[6], Michael J. Iadarola[2], Ashok B. Kulkarni[1,**]

[1]Functional Genomics Section, National Institute of Dental and Craniofacial Research, and [2]Department of Perioperative Medicine, Clinical Center, National Institutes of Health, Bethesda, MD, USA; [3]The School of Theoretical Modeling, Washington, DC, USA; [4]Institute of Genomics and Integrative Biology, New Delhi, India; [5]Neuronal Cytoskeletal Protein Regulation Section, National Institute of Neurological Disorders and Stroke, National Institutes of Health, Bethesda, MD, USA; [6]University of Maryland, School of Dentistry, Baltimore, MD, USA

*These authors contributed equally.

# Current address: Tulane University School of Medicine, New Orleans, LA, USA.

**Corresponding author

Supplementary Table 1. Putative CDK5 substrates in TRPV1 lineage DRG neurons

Human Gene	TRPV1 Lineage RPKM	non-TRPV1 Lineage RPKM	Fold > in TRPV1 Lineage	DRG/Sciatic	Pub.	MS	Predicted accessible	Best Position	Sequence			Score	
**TRPV1**	**157.9**	**1.39**	**113.60**	13.43	3	0	Cytoplasmic N-term	407	IAYSSSE	T	PNRHDML	0.4315	TRPV1 lineage enriched genes
DGKK	4.03	0.06	67.17	–	0	1	Intracellular protein	814	PEDINQT	S	PRRRSRR	0.2671	
CACNA1I	4.04	0.07	57.71	6.20	0	0	Cytoplasmic C-term	2148	PPPAPGL	T	PARKFSS	0.4082	
SLC17A8	8.25	0.27	30.56	3.50	0	0	Cytoplasmic C-term	541	LNHESFA	S	PKKKMSY	0.3296	
CRHR2	1.74	0.07	24.86	0.48	0	1	Cytoplasmic C-term	396	RAMSIPT	S	PTRISFH	0.5012	
**TRPA1**	**23.57**	**0.95**	**24.81**	**144.60**	0	0	Cytoplasmic N-term	448	SKSKDKK	S	PLHFAAS	0.4333	
LPAR3	45.84	1.91	24.00	2.43	0	0	Cytoplasmic loop	233	GSISRRR	T	PMKLMKT	0.4361	
AVPR1A	1.85	0.08	23.13	0.16	0	0	Cytoplasmic loop	79	VLLALHR	T	PRKTSRM	0.3454	
AVPR1A	–	–	–	–	0	5	Cytoplasmic C-term	†404	STGMWKD	S	PKSSKSI	0.5002	
KCNG2	7.05	0.4	17.63	8.48	0	0	Cytoplasmic N-term	134	EEERGEC	S	PKCRSLF	0.4418	
TNFAIP8L3	2.6	0.17	15.29	–	0	0	Intracellular protein	242	ELVQRHL	T	PRTHGRI	0.3587	
LPAR5	13.06	0.9	14.51	–	0	0	Cytoplasmic C-term	313	NTLRGLG	T	PHRARTS	0.3604	
KCNC2	9.92	0.7	14.17	63.58	0	0	Cytoplasmic N-term	78	QPSPPPL	S	PPPRAPP	0.41	
KCNH6	30.54	2.46	12.41	74.87	0	0	Cytoplasmic C-term	908	YGDLDDC	S	PKHRNSS	0.2979	
RGS14	4.46	0.41	10.88	1.16	0	2	Intracellular protein	456	ISKARDK	S	PCRSQGC	0.4577	
SCN11A	169.13	16.35	10.34	1013	0	0	Cytoplasmic loop	454	SLETSYF	T	PKKRKLF	0.2557	
TRPV3	2.24	0.31	7.23	0.43	0	0	Cytoplasmic N-term	35	EKRPAEI	T	PTKKSAH	0.4502	
EGR1	30.05	4.27	7.04	0.42	0	0	Intracellular protein	331	YPNRPSK	T	PPHERPY	0.2981	
EGR2*	3.32	8.38	0.40	0.29	0	0	Intracellular protein	333	YPNRPSK	T	PVHERPY	0.3476	
EGR3*	2.01	3.56	0.56	2.07	0	0	Intracellular protein	268	YPNRPSK	T	PLHERPH	0.3606	
SOCS3	14.65	2.16	6.78	0.66	0	0	Intracellular protein	159	SAQPLPG	S	PPRRAYY	0.2569	Additional selected genes
RGS3	37.76	6.5	5.81	4.75	0	0	Intracellular protein	496	TIPEESG	S	PSKGKSY	0.3867	
RGS3	–	–	–	–	0	0	Intracellular protein	124	RDEWTQT	S	PARKRIT	0.395	
RGS4	606.1	125.13	4.84	18.10	1	1	Intracellular protein	103	EEYKKIK	S	PSKLSPK	0.3942	
STMN1	356.54	79.1	4.51	0.37	43	239	Cytoplasmic N-term	38	SVPEFPL	S	PPKKKDL	0.3046	
HCN3	16.52	3.83	4.31	39.60	0	0	Cytoplasmic C-term	728	QRATGDG	S	PGRKGSG	0.3588	
SMARCA1	14.61	3.7	3.95	9.48	0	0	Intracellular protein	1045	KKKRATK	T	PMVKFSA	0.4912	
KCNN1	15.98	4.48	3.57	3.70	0	0	Cytoplasmic N-term	58	PARPSPG	S	PRGQPQD	0.4185	
BAHD1	23.52	7.13	3.30	–	0	1	Intracellular protein	746	PPSADYS	T	PPHRTVP	0.3445	
GRM7	24.72	8	3.09	90.54	0	5	Cytoplasmic C-term	900	CENVDPN	S	PAAKKKY	0.466	

†included because of large number of MS peptides; *Paralogs with the same site shown to demonstrate conserved domain

Supplementary Figure 1

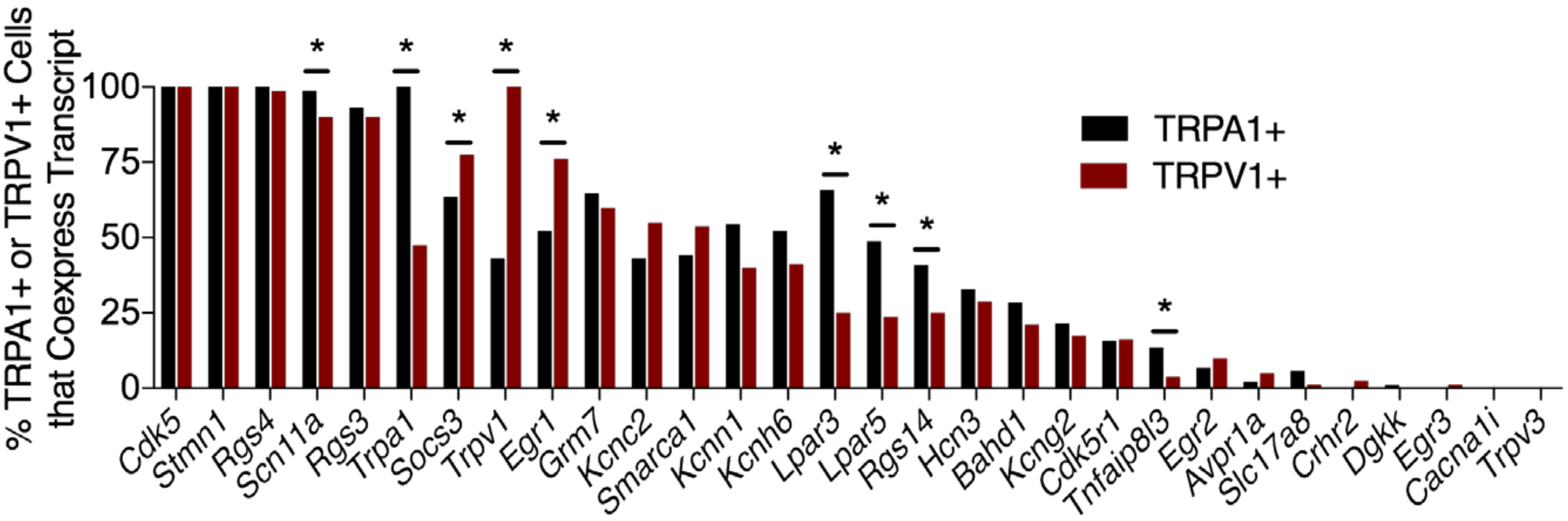

Supplementary Figure 2

```
TRPA1  26   GEDMDCSKESFKVDIEGDMCRLEDFIKNRRKLSKYEDENLCPLHHAAAEGQVELM-ELII  84
AnkB   28   GSKSDSNASFLRAARAGNLDKVVEYLKGGIDINTCNQNGLNALHLAAKEGHVGLVQELLG  87
                         R1                                R2

TRPA1  85   NGSSCEVLNIMDGYGNTPLHCAAEKNQVESVKFLLSQGANPNLRNRNMMSPLHIAVHGMY  144
AnkB   88   RGSS---VDSATKKGNTALHIASLAGQAEVVKVLVKEGANINAQSQNGFTPLYMAAQENH  144
                                   R3                               R4

TRPA1  145  NEVIKVLTEHKATNINLEGENGNTALMSTCAKDNSEALQILLEKGAKLCKSNKWGDYPVH  204
AnkB   145  IDVVKYLLENGA-NQSTATEDGFTPALVALQQGHNQAVAILLENDTK----GKVRLPALH  199
                                         R5

TRPA1  205  QAAFSGAKKCMELILAYGEKNGYSRETHINFVNHKKASPLHLAVQSGDLDMIKMCLDNGA  264
AnkB   200  IAARKDDTKSAALLLQNDHNADVQSKMMVNRTTESGFTPLHIAAHYGNVNVATLLLNRGA  259
                R6                                      R7

TRPA1  265  HIDMMENAKCMALHFAATQGATDIVKLMISSYTGSSDIVNAVDGNQETLLHRASLFDHHD  324
AnkB   260  AVDFTARNGITPLHVASKRGNTNMVKLLLD----RGGQIDAKTRDGLTPLHCAARSGHDQ  315
                             R8                                    R9

TRPA1  325  LAEYLISVGADINS  338
AnkB   316  VVELLKVVTEEVTT  329


TRPA1  247  AVQSGDLDMIKMCLDNGAHIDMMENAKCMALHFAATQGATDIVKLMISSYTGSSDIVNAV  306
AnkB   40   AARAGNLDKVVEYLKGGIDINTCNQNGLNALHLAAKEGHVGLVQELLGR--GSS--VDSA  95
                   R1.                           R2

TRPA1  307  DGNQETLLHRASLFDHHDLAEYLISVGADINSTDSEGRSPLILATASASWNIVNLLLCKG  366
AnkB   96   TKKGNTALHIASLAGQAEVVKVLVKEGANINAQSQNGFTPLYMAAQENHIDVVKYLLENG  155
                         R3                                R4

TRPA1  367  AKVDIKDHLGRNFLHLTVQQPYGLRNLRPEFMQMQHIKELVMDEDNDGCTPLHYACRQGV  426
AnkB   156  ANQSTAT--------------------------------------EDGFTPLAVALQQGH  177

TRPA1  427  PVSVNNLLGFNVSIHSKSKDKKSPLHFAASYGRINTCQRLLQ-----DISDTRLLNEGDL  481
AnkB   178  NQAVAILLEND----TKGKVRLPALHIAARKDDTKSAALLLQNDHNADVQSKMMVNRTTE  233
                    R5                     R6

TRPA1  482  HGMTPLHLAAKNGHDKVVQLLLKKGALF-LSDHNGWTALHHASMGGYTQTMKVILDTNLK  540
AnkB   234  SGFTPLHIAAHYGNVNVATLLLNRGAAVDFTARNGITPLHVASKRGNTNMVKLLLDRGGQ  293
                      R7                              R8

TRPA1  541  CTDRLDEEGNTALHFAAREGHAKAVAML  562
AnkB   294  -IDAKTRDGLTPLHCAARSGHDQVVELL  320
                            R9
```

Supplementary Figure 3

A

```
 61 EDENLCPLHHAAAEGQVELMELIINGSSCEVLNIM                      ANK1
 96 DGYGNTPLHCAAEKNQVESVKFLLSQGANPNLR                        ANK2
129 NRNMMSPLHIAVHGMYNEVIKVLTEHKATNINLE                       ANK3
163 GENGNTALMSTCAKDNSEALQILLEKGAKLCKS                        ANK4
196 NKWGDYPVHQAAFSGAKKCMELILAYGEKNGYSRETHINFV                ANK5
237 NHKKASPLHLAVQSGDLDMIKMCLDNGAHIDMM                        ANK6
270 ENAKCMALHFAATQGATDIVKLMISSYTGSSDIVNAV                    ANK7
307 DGNQETLLHRASLFDHHDLAEYLISVGADINST                        ANK8
340 DSEGRSPLILATASASWNIVNLLLCKGAKVDIK                        ANK9
373 DHLGRNFLHLTVQQPYGLRNLRPEFMQMQHIKELVM                     ANK10
411 DNDGCTPLHYACRQGVPVSVNNLLGFNVSIHSK                        ANK11
444 SKDKKSPLHFAASYGRINTCQRLLQDISDTRLLNEG                     ANK12
480 DLHGMTPLHLAAKNGHDKVVQLLLKKGALFLS                         ANK13
512 DHNGWTALHHASMGGYTQTMKVILDTNLKCTDRL                       ANK14
546 DEEGNTALHFAAREGHAKAVAMLLSYNADILL                         ANK15
578 NKKQASFLHIALHNKRKEVVLTTIRNKRWDECLQVFTH                   ANK16
616 NSPSNRCPIMEMVEYLPECMKVLLDFCMIPSTEDKS                     ANK17

151 PETGKTCLLKAmLNLHNGQNDTIALLLDVARKTDSLKQFVNASYTDS          ANK2/Trpv1
198 YYKGQTALHIAIERRNMTLVTLLVENGADVQAAANGDFFKK                ANK3/Trpv1
239 TKFGELPLSLAACTNQLAIVKFLLQNSWQPAKVVQLLLKKVKFLLQNSWQPA     ANK4/Trpv1
281 DSVGNTVLHALVEVADNTVDNTKFVTSmYNEILILGAKLHPTLKLEEIT        ANK5/Trpv1
330 NRKGLTPLALAASSGKIGVLAYILQREIHE                           ANK6/Trpv1

      xGxTPLHvAxxxGxxxyvxxLLxxGAxxxxxxx               consensus

      1234567890123456789012345678901234

        inner        outer
```

B

```
                    Ser242       Ser449        Thr485
                       ▽            ▽             ▽
            Human GKATPLHLAV | DKKSPLHFAAS | HGMTPLHLAA
              Cow GKASPLHMAV | DKKSPLHFAAS | HGMTPLHLAA
              Dog GKSSPLHMAV | DKKSPLHFAAS | HGMTPLHLAA
              Rat KKASPLHLAV | DKKSPLHFAAS | HGMTPLHLAA
            Mouse KKASPLHLAV | DKKSPLHFAAS | HGMTPLHLAA
          Chicken GKCSPLHLAV | DKKSPLHFAAS | KGMTPLHLAA
        Zebrafish SCSSPLHLAV | EKKSALHFAAE | KGLTPLHLAS
              Fly EGNVPLHSAV | NNESPLHFAAR | AGMTPLHISS
        Consensus .k.sPLHlAV | #kkSpLHFAAs | .G$TPLHlaa

            Human GKATPLHLAV | DKKSPLHFAAS | HGMTPLHLAA
              Fly EGNVPLHSAV | NNESPLHFAAR | AGMTPLHISS
       A.gambiae  EGNVPLHSAV | NNESPLHFAAR | EGLTPLHIAS
C.quinquefasciatus EGNVPLHSAV | NNESPLHFAAR | EGLTPLHIAS
       A.aegypti  (PARTIAL)  | NNESPLHFAAR | EGLTPLHIAS
        Consensus ....plh.av | #neSPLHFAAr | eG$TPLHias
```

C

```
Mouse      641 FCMIPSTEDKSCQDYHIEYNFKYLQCPLSMTKKVAPTQDV 680
Human      640 FCMLHSTEDKSCRDYYIEYNFKYLQCPLEFTKK-TPTQDV 678
Consensus      FCM  STEDKSC DY IEYNFKYLQCPL  TKK  PTQDV
```

Supplementary Figure 4

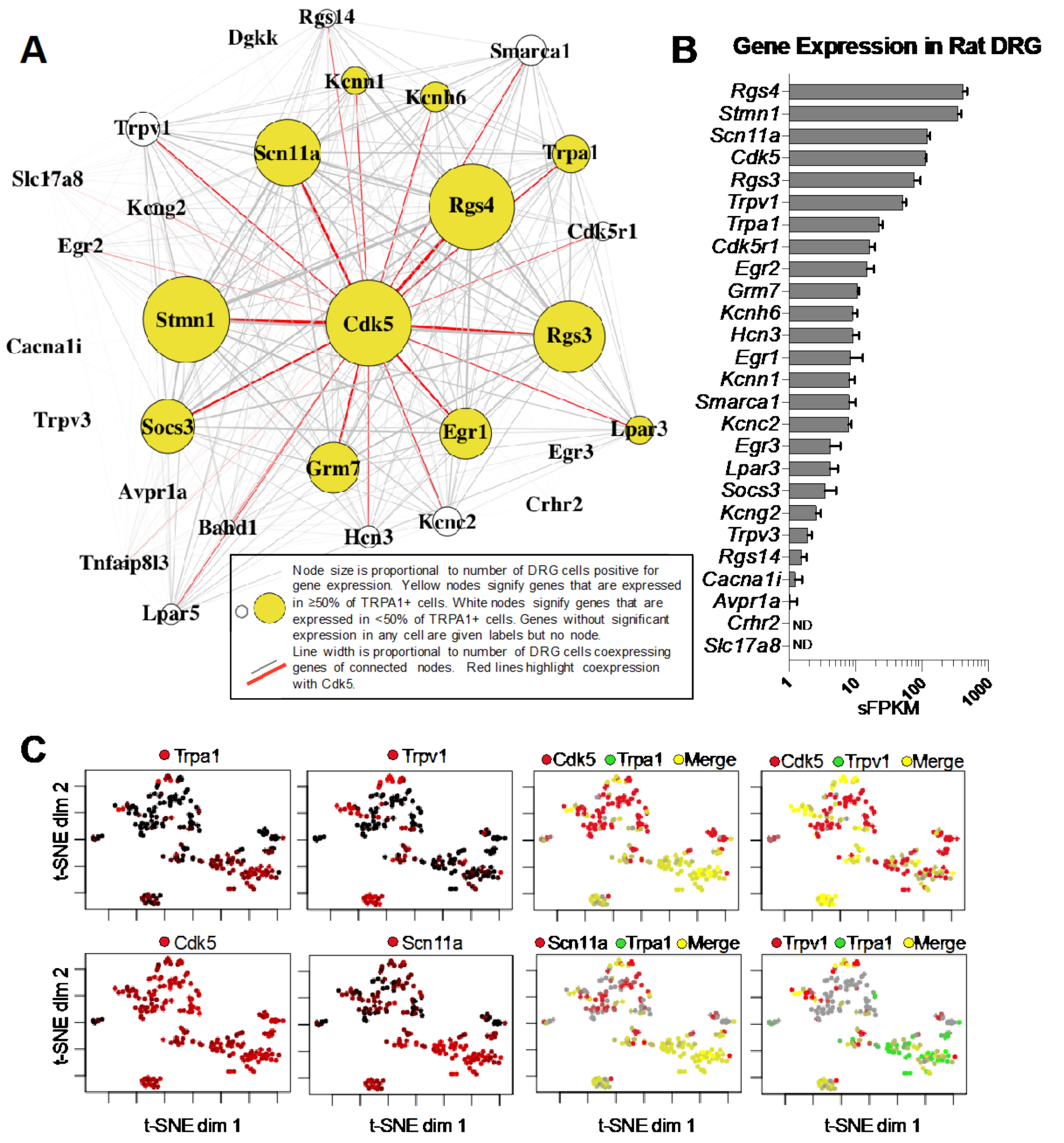

Supplementary Figure 5

A

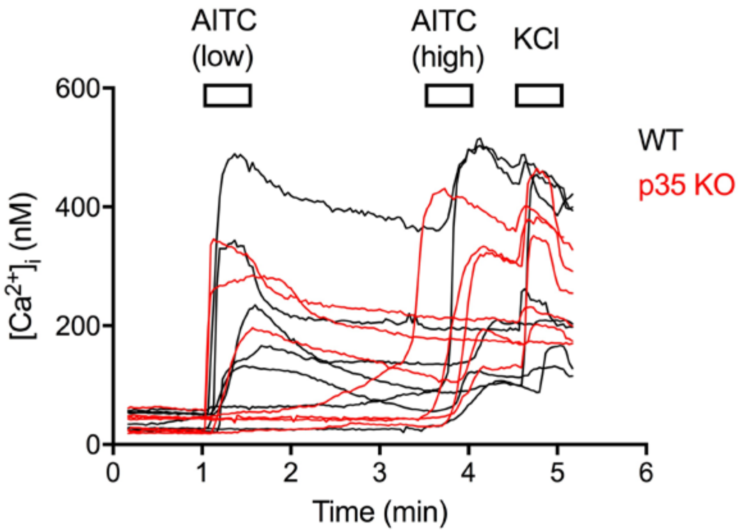

B

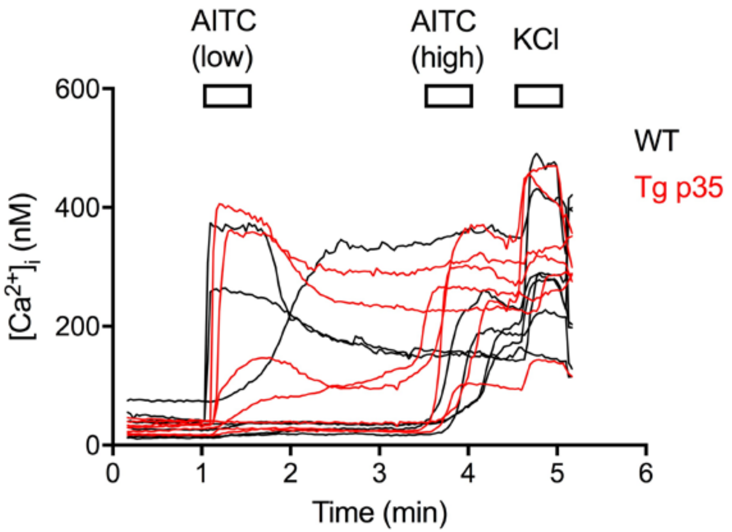

Supplement: Supplementary file 1 — Supplementary Information [file 41598_2018_19532_MOESM1_ESM.pdf]
